# Understanding Electron Transfer Reactions Using Constrained Density Functional Theory: Complications Due to Surface Interactions

**DOI:** 10.1021/acs.jpcc.2c06537

**Published:** 2023-02-09

**Authors:** Arsalan Hashemi, Pekka Peljo, Kari Laasonen

**Affiliations:** † Research Group of Computational Chemistry, Department of Chemistry and Materials Science, Aalto University, FI-00076 Aalto, Finland; ‡ Research Group of Battery Materials and Technologies, Department of Mechanical and Materials Engineering, Faculty of Technology, University of Turku, 20014 Turun Yliopisto, Finland

## Abstract

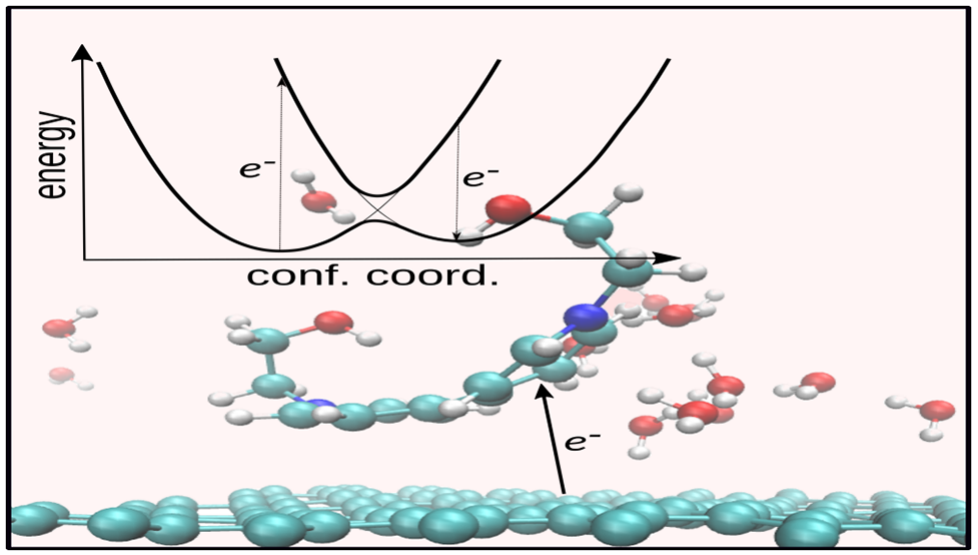

The kinetic rates of electrochemical reactions depend on electrodes and molecules in question. In a flow battery, where the electrolyte molecules are charged and discharged on the electrodes, the efficiency of the electron transfer is of crucial importance for the performance of the device. The purpose of this work is to present a systematic atomic-level computational protocol for studying electron transfer between electrolyte and electrode. The computations are done by using constrained density functional theory (CDFT) to ensure that the electron is either on the electrode or in the electrolyte. The ab initio molecular dynamics (AIMD) is used to simulate the movement of the atoms. We use the Marcus theory to predict electron transfer rates and the combined CDFT-AIMD approach to compute the parameters for the Marcus theory where it is needed. We model the electrode with a single layer of graphene and methylviologen, 4,4′-dimethyldiquat, desalted basic red 5, 2-hydroxy-1,4-naphthaquinone, and 1,1-di(2-ethanol)-4,4-bipyridinium were selected for the electrolyte molecules. All of these molecules undergo consecutive electrochemical reactions with one electron being transferred at each stage. Because of significant electrode–molecule interactions, it is not possible to evaluate outer-sphere ET. This theoretical study contributes toward the development of a realistic-level prediction of electron transfer kinetics suitable for energy storage applications.

## Introduction

Electron transfer (ET) is a key process in all redox reactions in (bio)chemistry,^1−3^ from natural photosynthesis to cellular respiration to electricity storage and conversion technologies.^4−11^ The importance of this area was highlighted by the 1992 Nobel Prize in Chemistry awarded to Rudolph Marcus “for his contributions to the theory of electron transfer reactions in chemical systems”. Understanding electron transfer reactions is also of crucial importance in selecting materials for flow batteries, a key candidate for stationary energy storage for storing wind and solar energy. In flow batteries, redox-active molecules typically dissolved in aqueous electrolyte solutions undergo electron transfer reactions with the electrodes in the electrochemical cell to store or discharge electricity. As the electrolytes are stored in large tanks and pumped through an electrochemical cell, the energy capacity and power density are decoupled. In other words, instead of storing electricity within the electrode itself, like conventional rechargeable batteries,^12,13^ energy is stored into solution inside the storage tanks, while power depends on the surface area of the electrochemical cell.

To minimize the voltage losses of the flow batteries, materials with facile electron transfer kinetics should be utilized. A temporary coupling between the electrode and the electrolyte occurs during the electrochemical redox reactions. These reactions take place at the electrode–electrolyte interface where the electrode provides a source or sink of electrons and the electrolyte carries redox-active species.^14−16^ The interaction at the interface is determined by the nature of the used materials. For a battery to perform well, the charge transfer between the electrode and the redox electrolyte must be fast. Generally, the efficiency of an electrochemical reaction is evaluated by the ET rate constant (*k*_ET_^0^).^17^ With this, it is possible to determine how fast electron transfers between the electroactive species and the electrode surfaces. Having a high *k*_ET_^0^ indicates fast system relaxation in response to ET and permits large current densities with low overpotentials. For this reason, it is important to determine the ET rate constant of the employed redox-active pairs when designing more efficient systems. While experimental techniques are well-established to examine electron transfer kinetic,^18,19^ two factors slow down the development of new redox systems. First, if one wishes to discover new redox-active species, their syntheses are complex processes that require substantial resources. Second, though, the basic principles of ET reactions are clear, we are not fully aware of the underlying processes at the microscopic level due to their high complexity. To help solve these problems, quantum-mechanical modeling can be used both to identify molecular properties and to rationalize kinetic differences.^20−22^ Recent studies by Martínez-González et al.^23,24^ have combined experiments with calculations to study the reduction reactions of a handful of organic molecules on the glassy carbon electrodes in different circumstances.

To simulate ET processes at the electrode–electrolyte interface, the electron density is separately localized on each counterpart. One group of atoms can be considered the electron acceptor while the other group is the electron donor. These two atomic groups also move at finite temperatures. Such a complete energetic picture of the ET reaction can be studied using the quantum-mechanical constrained density functional theory (CDFT) method^25−34^ in combination with the ab-initio molecular dynamics (AIMD) simulations. Finally, to assess the kinetics of the ET reactions, the Marcus theory^35^ can be used. All the parameters of the Marcus model will be computed using the CDFT-AIMD simulations.

Our group has presented an efficient CDFT implementation^36^ in the CP2K^37,38^ software. This code benefits (i) simultaneous inclusion of charge fragments in the CDFT-AIMD calculations to lower simulation wall clock time, (ii) reduction in computational time by efficient construction of the constraint weight function, (iii) solving electronic structure based on orbital transformation^39^ method in a three-tiered self-consistent field (SCF) approach, two for energy minimization and one for constraint Lagrangian maximization, and (iv) a hybrid Gaussian and plane-wave basis set^40^ to efficiently perform the simulations of low-symmetry systems. In the course of publishing this paper, we discovered a new paper that examined Hirshfeld charge partitioning rather than Becke’s.^41^

In this paper, a detailed protocol is presented to study ET between the solid-state electrode and the liquid electrolyte. We hope this paper paves the way for future computational studies, specifically on the mechanisms of ET redox reactions and screening organic molecules for storage applications.

## Methodology

### Differential ET Rate Constant

**Figure 1:**
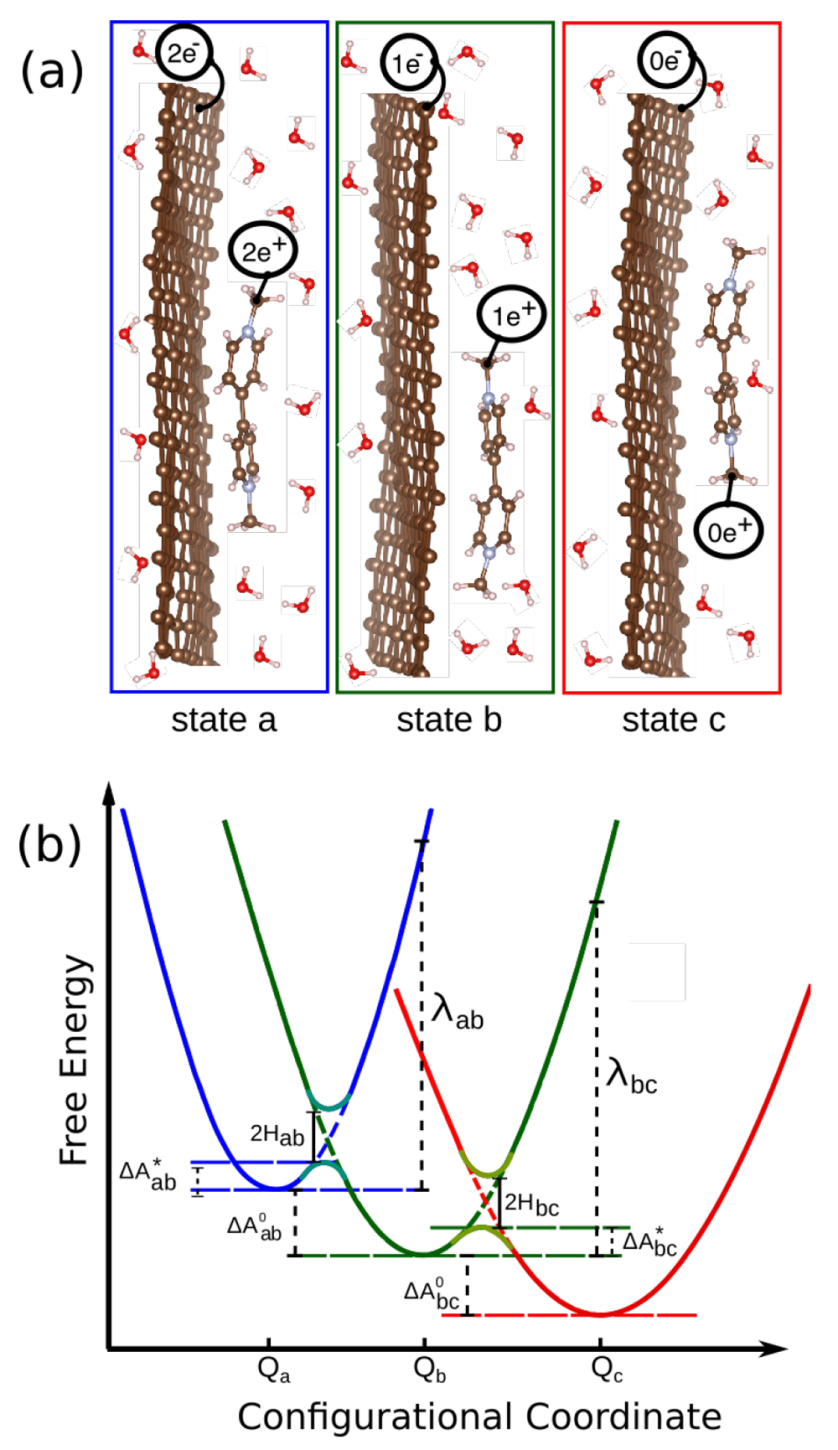
(a) Simulation boxes contain variously charged graphene and viologen molecule, solvated in water. Each box is representative of one state. The length of the model along the axis perpendicular to the graphene plane is schematic. (b) Schematic representation of potential energy curves versus nuclear (configurational) coordinate, which lumps together all the interatomic distances and collision angles, corresponding to the thermodynamic response to the electron transfer. The blue, green, and red parabolas represent different redox states.

Figure 1a shows a simplified view of three localized electronic states corresponding to three redox states as described in the Marcus theory (see Figure 1b). Because of the concept of temperature, the whole system fluctuates around equilibrium geometry in each state. Consequently, the free energy surface in each state is profiled by a quadratic curve based on the harmonic approximation.

The ETs occur sequentially in two steps here: two transferrable electrons are present at the electrode (state a), one electron is transferred to the electrolyte (state b), and the next electron is released from the electrode to the electrolyte (state c). It is critical to realize that two states involved in an ET reaction cross each other with similar curvatures.

Depending on how the atomic and electronic dynamics of the system and the medium interact, the ET reaction can be either diabatic or adiabatic. In the diabatic case, electron transfers from an initial electronic state, localized on the donor, to a final electronic state, localized on the acceptor, very rapidly (on time scales faster than nuclear motion). The system is then moved to its final state configuration by nuclear motions. The adiabatic reaction pathway forms a continuum of free energy between two states due to the strong electronic coupling effect.

The Marcus theory states that the diabatic rate constant, *k*_ET_ (1/s), is written as
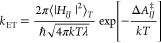
1Here, the diabatic electronic coupling at the crossing point, |*H*_*IJ*_|, has an exponential distance dependence:
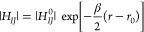
2where *H*_*IJ*_^0^ is the diabatic electronic coupling at the closest donor–acceptor separation distance *r* = *r*_0_ and β is the tunneling decay coefficient.^42,43^ The required energy to change the equilibrium configuration of the initial state (*I*) into the equilibrium configuration of the final state (*J*) while remaining on the same charge state as the initial one is called reorganization free energy, denoted by λ. Both reduction and oxidation processes are assumed to have the same λ value. In addition, ℏ, *k*, and ⟨...⟩_*T*_ are the reduced Planck constant, Boltzmann constant, and thermal averaging over nuclear configurations at temperature *T*, respectively. The activation barrier free energy Δ*A*_*IJ*_^‡^ at constant volume and temperature can be estimated as
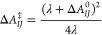
3where Δ*A*_*IJ*_^0^ is reaction free energy, i.e., the driving force for ET. In Figure 1b, these parameters were schematized.

The Marcus–Hush–Chidsey (MHC) theory is also commonly used to calculate heterogeneous outer-sphere ET. It includes electrode density of states (DOS) regardless of electrolyte chemistry, i.e., DOS is constant. Electrolytes are incorporated into ET through redox potential and reorganization energy obtained from a homogeneous medium. These critical factors at the electrode–electrolyte interface are affected by the donor–acceptor distance variation, but the MHC theory assumes that the electronic states of the system remain unchanged. On the other hand, the original Marcus theory accounts for direct interaction and instantaneous change in the reactants. In this work, we want to include these interactions. Listed below are references for readers interested in learning more about the MHC method.^44−47^

### Computational Details

Electronic structure calculations were performed within the framework of density functional theory (DFT) using the Gaussian plane-wave method (GPW) as implemented in the CP2K simulation software.^37,38^ The plane-wave and Gaussian basis sets were truncated with 350 and 35 Ry energy cutoffs, respectively. To present valence electrons, the optimized DZVP-MOLOPT-SR-GTH^48^ Gaussian basis sets were employed, whereas ionic cores were treated with norm-conserving GTH-PBE pseudopotentials.^49^ The calculations were performed with the Perdew–Burke–Ernzerhof^50^ (PBE) exchange-correlation functional. DFT-D3^51^ van der Waals (vdW) corrections with Becke–Johnson damping has been used throughout.^52^ Note that spin polarization was also included in the calculations.

The Becke weight function^53^ for real space partitioning was used to constrain electronic charges on each fragment. Spin states are not constrained. To avoid the poor scaling of the Becke method with the system size, the element-specific cutoff radii of 3.2, 2.5, 2.5, and 2.5 Å have been applied to C, N, H, and O atoms, respectively. We evaluated the robustness of CDFT with regard to the selection of cutoff values by keeping an eye on the energy gap values of molecules and the smoothness of charge density optimization convergence during CDFT-AIMD simulations. The Becke cell boundaries were shifted using element covalent radius:^54^ 0.76, 0.71, 0.31, and 0.66 Å for C, N, H, and O atoms, respectively. With this choice of atomic radius, we discovered meaningful values for the Becke charges on the O (≈ −0.38 *e*) and H (≈ +0.19 *e*) atoms in the neutral water molecules.^41^ The constrained charge convergence criterion was set to 10^–2^*e*. The systems were centered in cubic boxes with a 25 Å cell size in the *z*-axis direction. Periodic boundary conditions are applied to both static and dynamic calculations.

A two-step ET from graphene (donor) to molecule (acceptor) was modeled, as schematically shown in Figure 1a for a positively charged methylviologen^55,56^ (Me-Vi^2+^) molecule. There are three possible states for each molecule, in which graphene has a net charge of −2, −1, and 0. Normal DFT calculations (Table S1) show that the ground states for DMDQ, OH-Vi, and Me-Vi cases have +1 charge on molecules and −1 charge on graphene, while for dBR5 and 2HNQ, graphene has a charge of about −2 and the molecules are neutral. Overall, the studied electrochemical reactions can be summarized as follows:
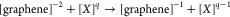
4a
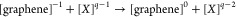
4bwhere *q* denotes the net charge of molecule X.

The total number of electrons is computed as 4*n*_C_ + 5*n*_N_ + 6*n*_O_ + *n*_H_ + *q* to constrain charges on a molecule. Here *n*_C_, *n*_N_, *n*_O_, and *n*_H_ are the number of carbon, nitrogen, oxygen, and hydrogen atoms in the molecule. A graphene containing 112 carbon atoms, on the other hand, can hold 448 electrons in its neutral state. There are 449 and 450 electrons in graphene, with net charges of −1 and −2, respectively.

**Figure 2:**
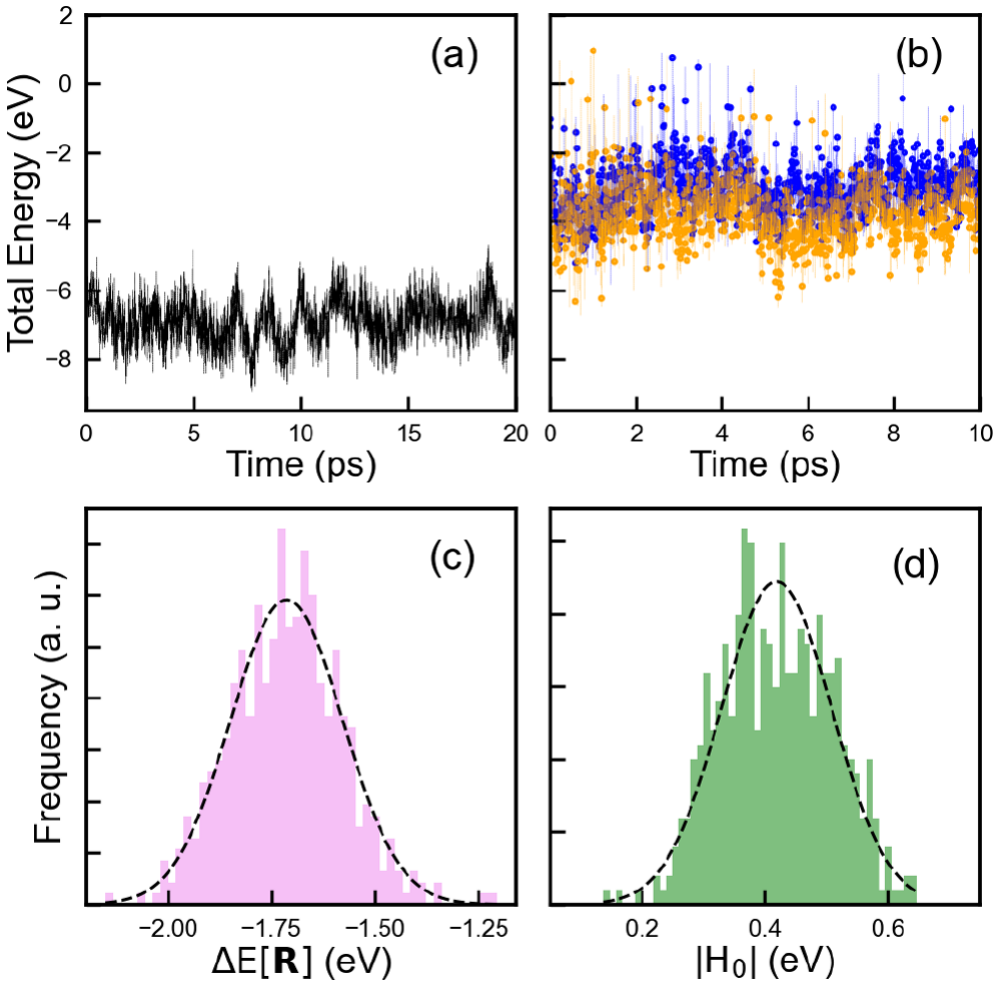
Time evolution of total energy during (a) AIMD and (b) CDFT-AIMD simulations of Me-Vi laid on graphene (≈12.35 Å × 12.82 Å) solvated by 40 water molecules. Blue and orange lines in subplot (b) illustrate +2 and +1 charge states on Me-Vi. Dots denote the taken snapshots for averaging. The normal distribution of (c) vertical energy gap and (d) diabatic electronic couplings for ET from graphene to Me-Vi^2+^. The Gaussian fit is represented by the dashed line for each case.

To evaluate the parameters of eq 1, knowledge of four points on the free energy surfaces is necessary.^57^ Two points are the energy of the *I* and *J* states in their respective local minima geometries. The other two points are obtained by computing the energy of the system accommodated at the equilibrium geometry of *I* while following the charge arrangement of *J*, and vice versa. To compute these quantities, our calculations were performed in three steps:

(i) For a periodic water-solvated complex system, AIMD was run for 20 ps in the canonical (NVT) ensemble at 300 K. Our setup contains no extra anions/cations. The time step was 0.5 fs. We rescaled velocity using the Bussi et al.^58^ thermostat with a target temperature of 330 K. By stabilizing atoms at the target temperature, this step will facilitate achieving an equilibrium configuration during CDFT-MD runs, which take place in the next stage. Figure 2a shows total energy versus time over the AIMD trajectory for the Me-Vi on graphene.

(ii) The well-equilibrated system was used to perform CDFT-AIMD calculations for 10 ps corresponding to the diabatic states *I* and *J*, defined based on the different charge constraints. After equilibration, 500 snapshots of atomic coordinates were taken (1 snapshot per 10 steps) from the last 2.5 ps of each trajectory. Note that there was no constraint on the charges of water molecules. Figure 2b shows the time evolution of the total energy during CDFT-AIMD simulations of Me-Vi^2+^ and Me-Vi^1+^ on graphene with charge −2 and −1, respectively.

(iii) For individual snapshots from the previous step, two single-shot and one mixed CDFT calculations were performed to evaluate the total energies and the diabatic electronic coupling. Finally, we compute λ and Δ*A*_*IJ*_^0^ as follows:^59,60^
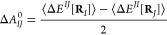
5
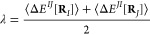
6where the vertical energy gaps are defined as
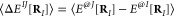
7
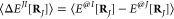
8The quantity *E*^@*I*^[**R**_*J*_] represents the total energy of the system with charge fragments of the state *I* but positioned at the nuclear coordinate of the state *J* energy minimum, i.e., **R**_*J*_. As shown in Figures 2c and 2d, the Gaussian distribution of the vertical energy gap and the electronic coupling can be specified by mean and standard deviation values. It should be noted that |*H*_*IJ*_^0^| is also calculated using the same formula as in the original study.^36^

When modeling the medium effects for the ET using molecular dynamics, it is important to account for the trade-off between computing power and simulation box size. We examine the effect of water molecule number on the Marcus parameters here. The rectangular unit cell of graphene, with the size of *a*_*x*_ = 2.469 and *a*_*y*_ = 4.274 Å, contains four carbon atoms. To investigate the optimum number of H_2_O, a supercell with a basal area of 12.35 × 12.82 Å^2^ (5 × 3 unit cell) was used. The water molecules were equally spread into the top and bottom of the graphene sheet.

The local structure of water molecules at the interface is influenced either by the solubility of redox-active molecules or by the hydrophobic nature of graphene. There is no doubt that the charge states of the used materials play an important role in determining these characteristics. For instance, the positively charged side of the water molecule attacks the negatively charged centers. In this manner, ETs cause solvent reorientation. The closer the water molecules are to the redox centers, the more strongly they reorient. In addition, those waters farther away from the redox centers are less affected. In order to capture these effects, regarding the low number of explicit water in our simulation, we additionally applied the self-consistent continuum solvation (SCCS) model^61^ via the dielectric constant ε_imp_ = 80. Note that we only add the implicit solvation model to the electronic structure calculations of the geometries derived from CDFT-AIMD calculations.

**Figure 3:**
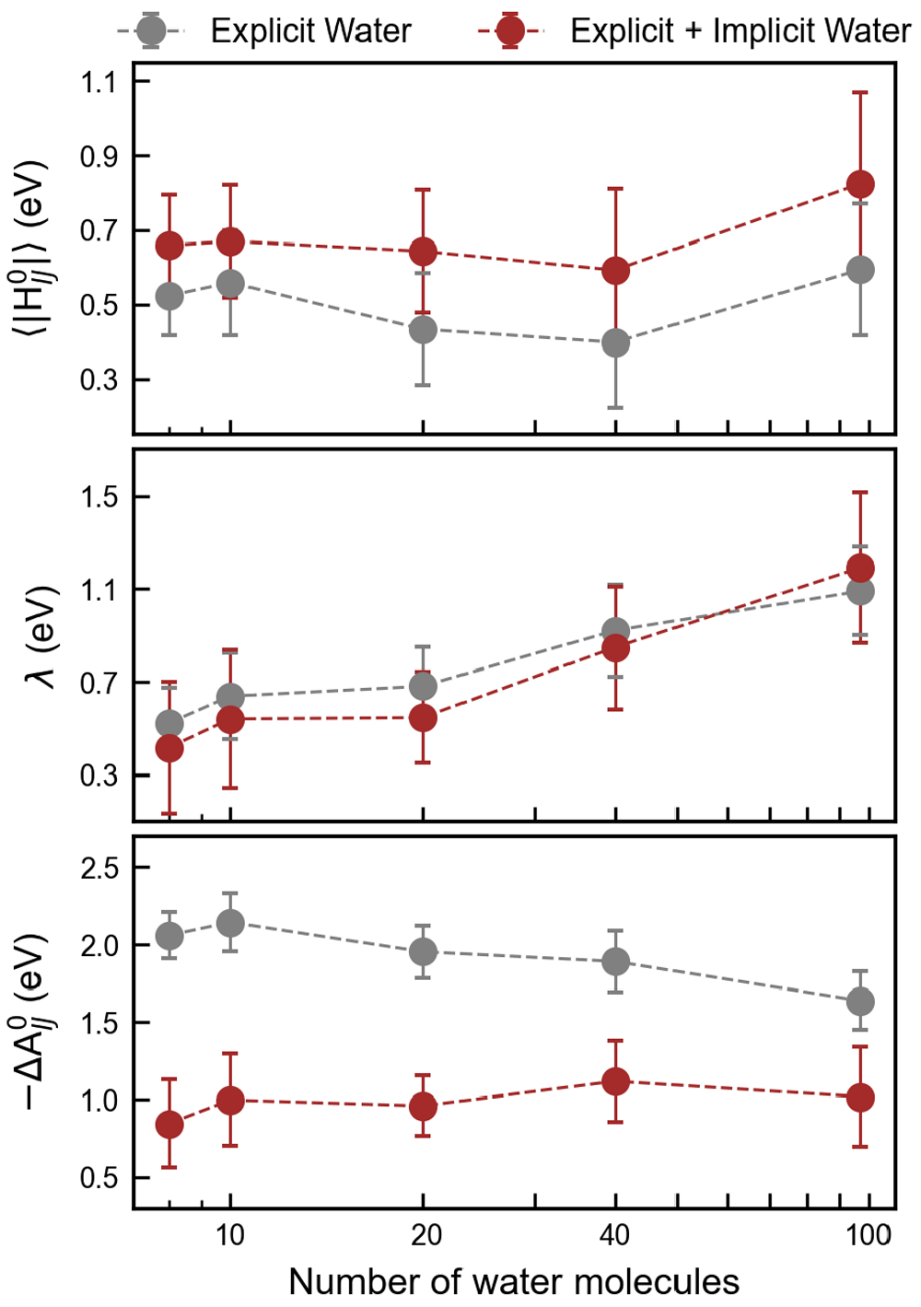
Diabatic electronic coupling |*H*_*IJ*_^0^| (top panel), the reorganization free energy λ_ab_ (middle panel), and reaction free energy Δ*A*_*IJ*_^0^ (bottom panel) for ET from double negatively charged graphene (5 × 3 unit cell) to double positively methyl viologen (Me-Vi^2+^) versus variation of water molecule density. Gray and brown dashed lines indicate the explicit and mixed explicit–implicit solvation models, respectively.

For the transfer of an electron from doubly negatively charged graphene to Me-Vi^2+^, all the Marcus parameters are computed as shown in Figure 3. We compare an explicit water solvent model with a combined explicit–implicit solvent model. A slightly larger wave function overlap between two interacting states results in a greater electronic coupling (*H*_*IJ*_^0^) in the explicit–implicit solvation model. The coupling remains, however, almost independent of the number of waters. It is clear that during ET chemical bonds relax and solvents reorient. Thus, total reorganization free energy can be divided into λ_in_ and λ_out_, which correspond to the bond (inner) and solvation (outer) effects, respectively. We see that the role of the explicit water (λ_out_) to stabilize the reorganization energy is crucial. The two models behave similarly. Finally, as the number of water molecules increases, the value of the ET free energy with the explicit solvation model gradually decreases, but the Δ*A*_*IJ*_^0^ value remains almost constant with the combined model. Even though the results are not fully converged, we see that the explicit–implicit solvation model is a better choice for this type of simulation.

Hereafter, we continue our calculations with 40 water molecules combined by the implicit SCCS as the solvent model. Aside from that to solvate all the molecules from our list on the same size graphene, we have to enlarge it to a 7 × 4 (17.28 × 17.09 Å^2^) supercell with the basal area of about 3 nm^2^. We provide open access to key geometries and input files for the simulated systems at 10.5281/zenodo.7388980.

## Results and Discussion

### Redox-Active Molecules

**Figure 4:**
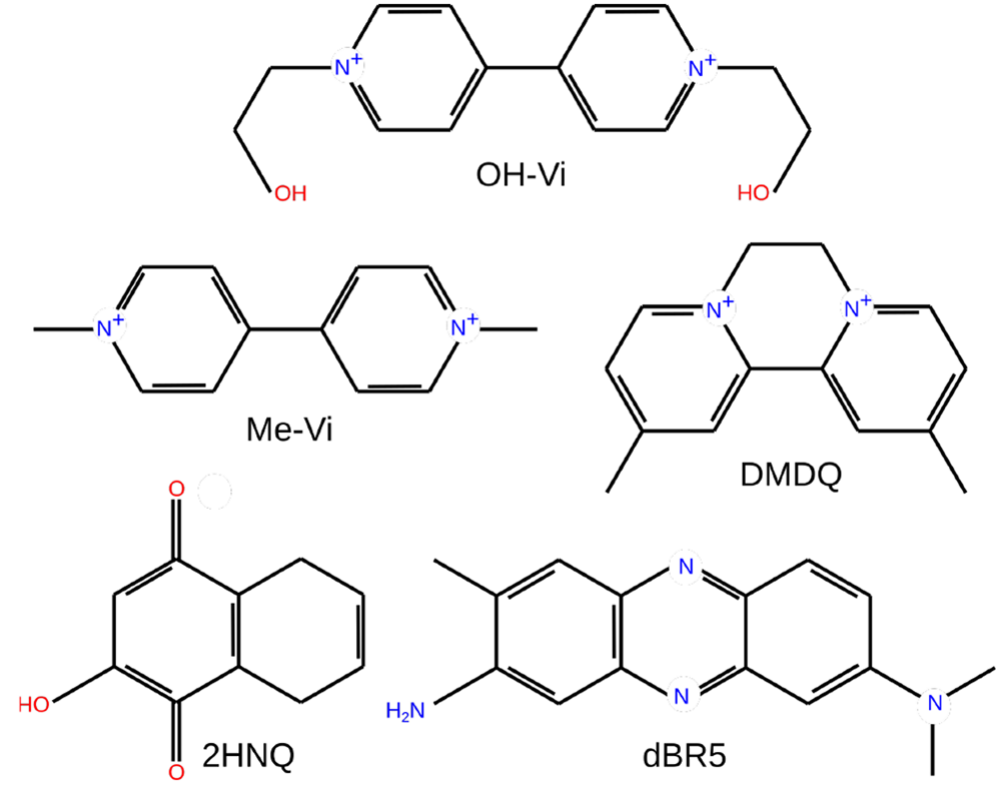
Structure of redox-active molecules in their oxidized forms.

Besides the Me-Vi molecule, we study 4,4′-dimethyldiquat^23^ (DMDQ), desalted Basic Red 5^62,63^ (dBR5), 2-hydroxy-1,4-naphthaquinone^64,65^ (2HNQ), and 1,1′-di(2-ethanol)-4,4′-bipyridinium^66,67^ (OH-Vi) molecules. The structures of these molecules are represented in Figure 4. All of these species have been tested as strong negolyte candidates in organic redox flow cells. Although Me-Vi, OH-Vi, and DMDQ are theoretically capable of a second ET, in practice they cannot dissolve in water after the reduction reactions.^23,55,56^ Their ability to work in nearly pH-neutral solutions can be one of their main advantages. In contrast, 2-HNQ and dBR5 both are protonated during the reduction reactions.^62,64,68^ Generally, reactions that include protons are slower and result in a lower electrochemical rate constant.

In order to understand the redox properties of the latter set of compounds, their mechanisms of reduction reactions can be described by ET, proton transfer (PT), and proton-coupled electron transfer (PCET) in the homogeneous solution phase,^69,70^ independent of the electrode interactions. As there are no physical electrodes and explicit solvents in this approach, thus, one must assess the cost of proton and electron participation in the redox reactions. Constants of an acid dissociation reaction^71^ and a redox reaction of an external electrode, such as the standard hydrogen electrode^72^ (SHE), serve as references for the electron and proton energy cost calculations, respectively. More technical information can be found in the Supporting Information. In this type of calculation, the traditional density functional theory (DFT) is used with an implicit solvation model, which is computationally more efficient than CDFT-AIMD. It should be noted that the local solvation surrounding the redox-active species may change at the interface, causing the acidity constant (p*K*_a_) to differ from the bulk.^73^ As a result, this effect may influence how the proton-coupled or detached ET at the electrode–electrolyte interface is described.

Figure S1 illustrates that at pH = 7, a nearly pH-neutral solution, the dBR5 molecule is fully reduced in the order of ET–PT–ET–PT reactions. The reduction reaction pathway of 2HNQ is ET–ET–PT–PT. At strong basic solutions, the protonation and electronation sequences can differ. For example, at pH = 14 only two electrons and one proton are involved in the reduction reaction of 2HNQ. These findings are in agreement with the experimental observations that measure the number of involved protons and electrons at a certain pH.^62−65^ However, protonation is inevitable during dBR5 and 2HNQ reductions. Despite its energetic origin, PCET will affect kinetics. Our DFT results show that for a sequential double-ET reduction, such as our CDFT simulations, an increase in applied electrochemical potential is required for the second ET. Again, the CDFT calculation does not take protonation into account and only considers ET.

### Marcus Theory Parameters from CDFT-AIMD

Marcus theory shows that it is possible for a reaction with a larger negative free energy (Δ*A*_*IJ*_^0^) to proceed more slowly than one with a less negative free energy. The region in which this occurs is called an ”inverted region” (see Figure S2a). A characteristic of this region is that the vertical gaps between the states of the reaction, *I* and *J*, have opposite signs. According to Table 1, all the reactions except the first ETs to the viologen- and diquat-based molecules are located in the inverted region. These reactions undergo a configuration coordinate expansion or compression up to the crossing point, followed by an inversion of motion.

**Table 1:** Calculated Energy-Related Parameters (eV) of the Marcus Theory Accompanied by Reduction Reaction Adiabatic Activation Barrier and Δ*A*_*IJ*_^ad^ Using AIMD-CDFT with 7 × 4 Graphene^a^

reaction	⟨Δ**E**^*IJ*^[**R**_*I*_] ⟩	⟨Δ**E**^*JI*^[**R**_*J*_] ⟩	Δ*A*_*IJ*_^0^	λ	Δ*A*_*IJ*_^‡^	⟨|*H*_*IJ*_^0^|⟩	Δ*A*_*IJ*_^*ad*^
DMDQ^+2/+1^	0.46 ± 0.15	0.80 ± 0.14	–0.17	0.63	0.08	0.58 ± 0.17	BL
DMDQ^+1/0^	1.89 ± 0.12	–0.59 ± 0.15	1.24	0.64	1.37	0.64 ± 0.09	0.73
OH-Vi^2+/1+^	0.40 ± 0.13	0.57 ± 0.14	–0.09	0.49	0.08	0.58 ± 0.18	BL
OH-Vi^+1/0^	2.27 ± 0.17	–0.59 ± 0.17	1.43	0.84	1.53	0.74 ± 0.09	0.79
Me-Vi^2+/1+^	0.16 ± 0.13	0.99 ± 0.22	–0.41	0.58	0.01	0.78 ± 0.21	BL
Me-Vi^+1/0^	1.72 ± 0.19	–0.68 ± 0.20	1.20	0.52	1.42	0.61 ± 0.09	0.81
dBR5^0/–1^	1.87 ± 0.21	–0.43 ± 0.19	1.15	0.72	1.21	0.41 ± 0.19	0.80
dBR5^–1/–2^	3.73 ± 0.17	–2.33 ± 0.25	3.03	0.70	4.96	0.64 ± 0.16	4.32
2HNQ^0/–1^	–0.06 ± 0.14	0.88 ± 0.17	–0.47	0.41	0.00	0.73 ± 0.23	BL
2HNQ^–1/–2^	2.88 ± 0.28	–0.60 ± 0.18	1.74	1.14	1.81	1.08 ± 0.15	0.73

a BL indicates adiabatically barrierless reactions. Standard deviation values are given for averaged vertical gap energy and diabatic coupling.

Between the two states, the configuration coordinate can also be continuously expanded or compressed. In this case, the electrochemical reaction takes place in a “normal region” (see Figure S2b). Here, thermodynamics and kinetics are directly related; i.e., a reaction with a larger negative Δ*A*_*IJ*_^0^ is faster. Both vertical gaps are also positive. Of the reactions, only DMDQ^+2/+1^, OH-Vi^2+/1+^, and Me-Vi^2+/1+^ exist in the normal Marcus region. An electron hopping along the vertical gap releases energy if Δ*E*^*IJ*^[**R**_*I*_] is negative. Therefore, in the 2HNQ^0/–1^ reaction, the electron can spontaneously jump from the ground state *I* to the excited state *J*, while the whole system fluctuates around the minima of the state *I* (**R**_*I*_). Overall, due to the increase of the donor–acceptor distance and the lower tendency of the molecules to accept the second electron, there is a systematic increase of Δ*E*^*IJ*^[**R**_*I*_].

The Δ*A*_*IJ*_^0^ value indicates the direction of a spontaneous electrochemical reaction. It is negative when the reduction reaction, i.e., the ET from graphene to the molecule, is energetically favorable. It appears that only the first ET from doubly negatively charged graphene to the individual DMDQ^+2^, OH-Vi^2+^, Me-Vi^2+^, and 2HNQ^0^ molecules are thermodynamically favorable. Clearly, the energy level of the electrode, which is adjustable by the externally applied potential, plays an important role in the interactions between electrode and electrolyte.^74^ For example, one can apply a negative potential to shift the parabola of the product downward to favor the ET reactions.

The reorganization free energies (λ), representative of the stiffness of systems, include both the rigidity of the interatomic bonds and the reorientation of the water molecules. Compared to the calculated data in ref (23) for the Me-Vi and DMDQ molecules, our results show larger values due to the presence of explicit water. Our study showed that the total reorganization energies are strongly influenced by the way water coordinates toward the redox couples (see Figure 3). When molecules are located at the closest distance from the electrode, the strong interaction between the molecules and the out-of-plane vibrations of graphene leads to smaller λ values.^75^ As shown in Figure S3, the partial insertion of a water molecule between neutral graphene and OH-Vi^0^ leads to an increase in the distance and consequently a non-negligible enhancement in λ during the second ET. Compared to the 2HNQ^0/–1^ reaction, 2HNQ^–1/–2^ shows a symmetrical increase in the averaged distance from graphene (about 1.3 Å more). This results in an increase in λ to 1.14 eV.

The diabatic activation free energy, Δ*A*_*IJ*_^‡^, varies greatly: the DMDQ^+2/+1^, OH-Vi^2+/1+^, Me-Vi^2+/1+^, and 2HNQ^0/–1^ reactions are nearly barrierless, the dBR5^–1/–2^ reaction has a substantial barrier, and the remainders are in the middle. When compared to the first ETs, all of the second ETs exhibit a large increase in barrier energy. dBR5 becomes very nucleophile after the first ET. This makes it difficult to add the second electron, leading to an unreachable activation barrier. The activation barrier free energy for dBR5^–1/–2^ emphasizes the possibility for protonation after the first ET.

Diabatic electronic coupling (|*H*_*IJ*_^0^|) mainly affects the activation barrier. Considering splitting, we can reasonably define the adiabatic activation barrier Δ*A*_*IJ*_^ad^ as^76^
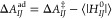
Clearly, a stronger |*H*_*IJ*_^0^| lowers the reaction barrier and allows the reaction to proceed adiabatically. Our calculations predict large ⟨|*H*_*IJ*_^0^|⟩ values ranging from 0.4 to 1.1 eV. Hence, we interpret that these amplitudes of couplings suggest adiabatic ET reactions. It is important to note that the coupling matrix is quite sensitive to computational details. For instance, prior research conducted by our team examined the exchange-correlational functional effect.^36^ In the hybrid functional instances, the average diabatic coupling estimated using PBE is around 5 times PBE0.^77^ This has been addressed to the more localized wave functions of two charge states in PBE0. In this study, our PBE0 calculations did not converge, and we use the PBE numbers. To estimate the effect of weaker PBE0 coupling, we scaled the |*H*_*IJ*_^0^| values by 0.2. This did not qualitatively change the results of Δ*A*_*IJ*_^ad.^.

We find that both the fast and slow ETs are possible for the studied redox reactions. The barrierless ET reactions occur when molecules are strongly bound to graphene. Our results are validated by the existing experiments,^23,24^ where strong adsorption of active molecules to electrodes has been confirmed for DMDQ^+2/+1^ and Me-Vi^2+/1+^ reactions. On the free energy surface, the minima for the reactant and product states merge into a single deep minimum in this class (see Figure S2c). In another class, the slow ETs have an energy barrier of <0.81 eV, except for dBR5^–1/–2^. The electron is displaced along a double-minimum surface (see Figure S2d).

### Finite-Size Effects

In a reduction reaction, electrons transfer from an electrode to a redox species when the Fermi level of the electrode exceeds the energy level of the electrons in the redox species.^78−80^ The electrode potential determines the cost of electrons sourced for the reactions. Thus, Fermi levels and electrode electronic structures are crucial for the performance of electrochemical systems (since electrochemical reactions are driven by potentials at the electrode surface). It is realistic to assume that transferring a handful of electrons during electrochemical reactions will not significantly change the electrode potential (the electrode’s Fermi level remains nearly constant due to the externally applied potential and also the electrode’s nature).

The problem of a constant potential is extremely challenging in computational studies because of the electrode “finite-size effect”.^81−83^ Similarly, our CDFT-AIMD simulations using 7 × 4 graphene shows dramatic changes in the Fermi level: for charge states of −2, −1, and 0, the levels are positioned at −0.79, −2.84, and −3.79 eV, respectively. In order to solve this problem, we developed a model by increasing the size of graphene. Figure S4a illustrates a large variation in the Fermi level energy in smaller supercells with respect to their charge state. We find that the variation drops as a function of 1/*N* , where *N* is the number of carbon atoms in the supercell. It is therefore necessary to have an infinite size for the electrode to remain at a constant potential. However, we use a supercell of 37.04 Å × 38.47 Å (15 × 9 unit cell that contains 540 carbon atoms) whose Fermi level energy varies <130 meV between two consecutive charge states to make our calculation feasible.

Clearly, a full AIMD-CDFT simulation of the complex electrode–electrolyte interface is extremely costly for such a large supercell. To solve this problem, (i) 25 geometries from our previous AIMD-CDFT calculations for the 7 × 4 graphene supercell are randomly selected, (ii) 15 × 9 graphene supercells are generated by embedding carbon atoms into the graphene from the previous step (see Figure S5), and (iii) while the atoms from step i are frozen, the geometry of the added carbon atoms from step ii is optimized using DFT. The implicit SCCS solvation model with the dielectric constant of water is applied to the entire supercell for further calculations.

Now all the Marcus parameters can be recalculated for an electrode with nearly constant potential (tabulated in Table 2). Qualitatively, Figures S4b and S4c show a systematic increase and decrease in the Δ*A*_*IJ*_^0^ and λ values, respectively. Consequently, activation barriers rise, which results in slower kinetics. For the larger supercell, the electronic couplings were slightly reduced due to a more delocalized net charge compared to the surface of 7 × 4 graphene supercell. Nevertheless, the picture of the energy surface of each reaction remains similar.

**Table 2:** Calculated Energy-Related Parameters (eV) of the Marcus Theory Using the 15 × 9 System^a^

reaction	⟨Δ**E**^*IJ*^[**R**_*I*_]⟩	⟨Δ**E**^*JI*^[**R**_*J*_]⟩	Δ*A*_*IJ*_^0^	λ	Δ*A*_*IJ*_^‡^	⟨|*H*_*IJ*_^0^|⟩	–*eE*_sol_^0^
DMDQ^+2/+1^	0.69 ± 0.29	0.23 ± 0.23	0.23	0.46	0.26	0.52 ± 0.11	–0.19
DMDQ^+1/0^	1.94 ± 0.28	–0.88 ± 0.18	1.24	0.53	1.77	0.23 ± 0.07	0.57
OH-Vi^2+/1+^	0.87 ± 0.17	0.04 ± 0.11	0.42	0.46	0.42	0.51 ± 0.10	–0.32
OH-Vi^+1/0^	2.32 ± 0.28	–0.81 ± 0.35	1.57	0.75	1.78	0.37 ± 0.08	0.41
Me-Vi^2+/1+^	0.56 ± 0.16	0.29 ± 0.13	0.14	0.43	0.18	0.88 ± 0.13	–0.29
Me-Vi^+1/0^	1.80 ± 0.28	–1.01 ± 0.25	1.40	0.40	2.05	0.25 ± 0.10	0.45
dBR5^0/–1^	2.50 ± 0.13	–1.32 ± 0.19	1.91	0.59	2.65	0.47 ± 0.15	0.83
dBR5^–1/–2^	3.87 ± 0.19	–2.81 ± 0.31	3.34	0.53	7.06	0.43 ± 0.16	1.52
2HNQ^0/–1^	0.61 ± 0.22	–0.06 ± 0.27	0.34	0.28	0.34	0.73 ± 0.07	–0.31
2HNQ^–1/–2^	2.84 ± 0.26	–1.01 ± 0.30	1.92	0.91	2.20	0.67 ± 0.11	0.55

a A standard hydrogen electrode (SHE) is used as the reference point for the redox reaction potentials in the solution phase, *E*_sol_^0^.

We also compute the diabatic electron transfer rates (*k*_ET_) for the reactions, even though the strong coupling mostly motivates the adiabatic electron transfer path. In the absence of applied potentials and for molecules located closest to the electrode, low barrier reactions, those Δ*A*_*IJ*_^0^ < 0.42, are extremely fast, while the remainders are very slow. When the potential wells of state *I* and state *J* become symmetric by applying an external potential, i.e., when −Δ*A*_*IJ*_^0^ = 0, the barrier for each reaction is λ/4. Consequently, the ET rate is defined by the rate constant (*k*_ET_^0^). Our calculated *k*_ET_^0^ values are very high due to the low reorganization energies caused by the small donor–acceptor distances. In some cases, we might not be able to use such an external potential because we move outside the practical water potential window of ca. −1.5 to +1.5 V.^84^ It is under this condition that electrochemical systems are prone to side reactions such as hydrogen evolution reactions and oxygen evolution reactions, which result in low performance.

### Potentials

There are three types of potentials in our study: (i) externally applied potential (*E*_appl._) that affects to the electrode’s Fermi level energy, (ii) solution phase onset potential (*E*_sol_^0^) that equals to free energy change of redox reaction divided by electron charge, and (iii) interfacial electrochemical reaction potential *E*_*IJ*_^0^ = −Δ*A*_*IJ*_^0^/*e*. These three components can be related by
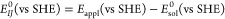
9where, typically, the standard hydrogen electrode (SHE) is defined as the reference electrode.

The ET rate constant is measured at an equilibrium state where the Gibbs free energies of the products equal those of the reactants, i.e., *E*_*IJ*_^0^ = 0. In addition, the electron energy in the solution (molecule’s LUMO energy) must equilibrate with the Fermi level in the electrode at the instance of ET.^85^ When an electrode–electrolyte combination is determined, *E*_appl_ is the only tool available to an operator to reach an equilibrium state. Suppose Δ*A*_*IJ*_^0^ > 0, a negative value of *E*_appl_ is applied to the electrode to upshift the Fermi level. Hence, it minimizes the Gibbs free energy difference.

There is no control over the *E*_appl._ potential in our study, and it is subject to change depending on the molecule that is active at the interface. Before taking any further action, it is necessary to consider two points. First, graphene is the reference electrode for *E*_*IJ*_^0^ values, not SHE. There is an *E*_ref_^gr,X^ term which shifts the reaction potential to the SHE reference for each molecule X. Thus, eq 9 can be rewritten as
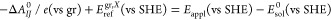
10Second, the implicit solvent model is used to simulate the formal reduction potential *E*_sol_^0^ without the presence of physical electrodes and electrolytes (see the Supporting Information for more details.). This means that the calculations were conducted at a different level than in CDFT-AIMD. However, it has been well-established that the employed approach for *E*_sol_^0^ calculations provides reliable results when compared to experiments.^86^ Indeed, there is strong agreement between our results and experimental data wherever it exists.

Once again *E*_sol_^0^ data show that it is thermodynamically favorable for the DMDQ^+2^, OH-Vi^2+^, Me-Vi^2+^, and 2HNQ^0^ molecules to undergo the first ET reduction reactions. The strong electron-accepting nature of dications leads to a larger *E*_sol_^0^ than the 2HNQ^0^ molecule. A negative redox potential of about −0.83 V_SHE_ is required for dBR5°, which is compensated by the electrode. As expected, the second ET reactions require negative potentials. There is only the dBR5^–1/–2^ reduction reaction that requires a potential of −1.50 V_SHE_ that is beyond the permitted range and results in a detrimental hydrogen evolution reaction.^87^ These findings are supported by CDFT-AIMD results. We therefore interpret that the computationally cheaper *E*_sol_^0^ is a powerful descriptor of redox properties for high-throughput screening.

**Figure 5:**
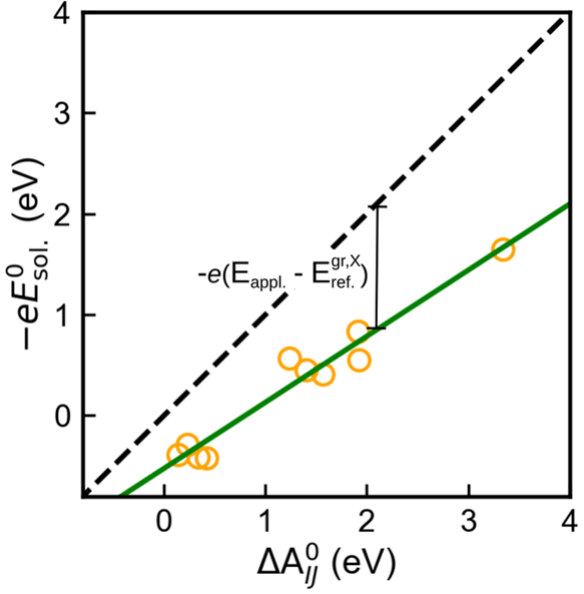
Reduction reaction free energies of various organic compounds in the homogeneous solution phase (−*eE*_sol_^0^) versus heterogeneous electrode–electrolyte phase (Δ*A*_*IJ*_^0^). *E*_appl_ and *E*_ref_^gr^ denote the externally applied potential to the electrode–electrolyte interface by the electrode and the shift of graphene electrode’s potential with respect to the reference standard hydrogen electrode, respectively. Fitting a linear regression model (green line) to data (orange circles).

All reactions in our CDFT-AIMD were studied in nonequilibrium states. When we compare *E*_*IJ*_^0^ and *E*_sol_^0^, we find the same trend in the energy values. According to eq 10, the difference comes from *E*_appl._ and *E*_ref_^gr,X^ terms. As shown in Figure 5, the deviation between these two terms slightly increases at lower reaction potential.

## Concluding Remarks

In this paper, the CDFT-AIMD methodology for simulating the electron transfer process across an electrode–electrolyte interface was applied. We have studied the kinetics of electron transfer from graphene to a series of redox-active molecules using Marcus theory. This approach allows us to not only assess the transfer rate of electrons more realistically but also to gain insights into the chemistry of redox reactions in the aqueous systems. A large electrode and explicit solvent were essential for modeling electrode–electrolyte interface systems and evaluating Marcus parameters in our study.

Our examination of the redox reactions revealed a few things. First, the electronic couplings were very strong, making adiabatic pathways more likely. Second, due to electronic couplings and small absolute values of the reaction free energies, both single-minimum and double-minimum Gibbs free energy surfaces were observed for the electron transfer processes. Accordingly, we would expect strong and weak bindings between the electrode and molecule in the former case and the latter case, respectively.

Even though the current simulations have provided valuable insight into the kinetics and thermodynamics of electrochemical reactions on a microscopic level, the electron transfer rate constant values do not match the experiments. This discrepancy is caused by Marcus theory failure due to strong electronic coupling, but the parameters themselves are accurate.

Now that we have tools for studying electron transfer, it indeed seems necessary to rethink the model system. As an example, even though electrode contamination is not of interest for flow battery devices, due to the strong adsorption of active molecules onto the electrode surfaces, we may be able to use a combined electrode–molecule system as an electrode model. As another example, distance-dependent electron reactions should be investigated for a more realistic level of study. The electron can tunnel some distance away from the electrode. Our future studies will address them.

As a final note, this simulation protocol also opens up new computational research opportunities in the field of electrochemical sensors, where solid-state transducers are paired with active materials.

## Supporting Information Available

A theoretical extraction of Marcus theory and a computational approach to solution-phase redox potential are presented in Figures S1–S5 (PDF)
