# Supplementary material for: Understanding Electron Transfer Reactions Using Constrained Density Functional Theory: Complications Due to Surface Interactions
Source: J Phys Chem C Nanomater Interfaces. 2023 Feb 9;127(7):3398–407. doi: 10.1021/acs.jpcc.2c06537 (PMC9969872; doi:10.1021/acs.jpcc.2c06537)
Supplement: Supplementary file 1 — jp2c06537_si_001.pdf [file jp2c06537_si_001.pdf]

Understanding Electron Transfer Reactions using Constrained Density Functional Theory: Complications due to Surface Interactions

Arsalan Hashemi,[†] Pekka Peljo,[‡] and Kari Laasonen[*,†]

†*Research Group of Computational Chemistry, Department of Chemistry and Materials Science, Aalto University, FI-00076 Aalto, Finland*

‡*Research Group of Battery Materials and Technologies, Department of Mechanical and Materials Engineering, Faculty of Technology, University of Turku, 20014 Turun Yliopisto, Finland*

E-mail: kari.laasonen@aalto.fi

Contents

1 Diabatic and adiabatic electron transfer rates **S2**

2 Normal DFT and Lowdin charge analysis **S4**

3 Redox potential in solution (homogeneous) phase **S5**

4 Marcus theory regions and adiabatic free energy surfaces **S8**

5 Snapshot taken along the CDFT-AIMD **S9**

6 Finite-size effects and scheme of supercell extension **S9**

1 Diabatic and adiabatic electron transfer rates

For a two-state system, the electron transfer rate constant (k_{ET}) is defined as

$$k_{ET} = \nu_n k_{el} k_n, \tag{S1}$$

where ν_n and k_n indicate nuclear vibration frequency and nuclear reorganization factor. In addition, k_{el} is electronic transmission coefficient defines as

$$k_{el} = \frac{2P_{IJ}}{1 + P_{IJ}}. \tag{S2}$$

It indicates the likelihood of an electron transfer taking place between a molecular acceptor and donor, or a transition (crossing) from the initial state I to the final state J. When the energy surfaces are parabolic and identical in shape, the Landau-Zener expression can be used to define P_{IJ}. When the energy surfaces are parabolic and identical in shape can be defined by the Landau-Zener expression:[1,2]

$$P_{IJ} = 1 - \exp[-\frac{\nu_{el}}{2\nu_n}], \tag{S3}$$

where

$$\nu_{el} = \frac{4\pi^2|H_{IJ}|^2}{h\sqrt{4\pi\lambda k_{\mathrm{B}}T}}, \tag{S4}$$

ν_{el} (S^{-1}) is the electron hopping frequency in the intersection region at ta donor-acceptor separation r (Å), H_{IJ} is the diabatic electronic coupling at separation distance r, λ is the reorganization energy, finally, h, k_B, and T Planck's constant, Boltzmann constant, and temperature, respectively.

The electronic coupling element, H_{IJ}, has an exponential distance dependence

$$H_{IJ} = H_{IJ}^0 \exp[-\frac{\beta}{2}(r - r_0)] \tag{S5}$$

Taken together all the above equations, one can write

$$P_{IJ} = 1 - \exp[-\frac{\nu_{el}^0 \exp[-\beta(\mathrm{r} - \mathrm{r}_0)]}{2}], \tag{S6}$$

where, ν_{el}^0, the value of ν_{el} when $r = r_0$, is defined as

$$\nu_{el}^0 = \frac{4\pi^2 |H_{IJ}^0|^2}{h\sqrt{4\pi\lambda k_\mathrm{B}T}} \tag{S7}$$

Combing Eqs. (S2) and (S6), one obtains

$$k_{el} = \frac{2(1 - \exp[-\frac{\nu_{el}^0}{2\nu_n}\exp[-\beta(\mathrm{r} - \mathrm{r}_0)]])}{2 - \exp[-\frac{\nu_{el}^0}{2\nu_n}\exp[-\beta(\mathrm{r} - \mathrm{r}_0)]]}. \tag{S8}$$

The analytical simplification of Eq. (S8) occurs based on two assumptions:

- When $\frac{\nu_{el}^0}{2\nu_n}\exp[-\beta(\mathrm{r} - \mathrm{r}_0)] << 1$. This condition will be met for (i) outer-sphere ET ($\exp[-\beta(\mathrm{r} - \mathrm{r}_0)] << 1$) and (ii) nonadiabatic ET ($\frac{\nu_{el}^0}{2\nu_n} << 1$). Using Taylor series expansion, we obtain

$$\exp[\frac{\nu_\mathrm{el}^0}{2\nu_\mathrm{n}}\exp[-\beta(\mathrm{r} - \mathrm{r}_0)]] \approx 1 - \frac{\nu_\mathrm{el}^0}{2\nu_\mathrm{n}}\exp[-\beta(\mathrm{r} - \mathrm{r}_0)]. \tag{S9}$$

  Thus,

$$k_{el} = \frac{4\pi^2 |H_{IJ}|^2 \exp[-\beta(r - r_0)]}{h\nu_n\sqrt{4\pi\lambda k_\mathrm{B}T}}. \tag{S10}$$

  Finally, one can write

$$k_{ET} = k_n \nu_{el}^0 \exp[-\beta(r - r_0)] \tag{S11}$$

- On the other hand, if $\frac{\nu_{el}^0}{2\nu_n}\exp[-\beta(\mathrm{r} - \mathrm{r}_0)] >> 1$, then

$$\exp[\frac{\nu_\mathrm{el}^0}{2\nu_\mathrm{n}}\exp[-\beta(\mathrm{r} - \mathrm{r}_0)]] \approx 0. \tag{S12}$$

Similarly, the ET will be adiabatic or inner-sphere with:

$$k_{ET} = \nu_n k_n. \tag{S13}$$

2 Normal DFT and Lowdin charge analysis

Partial atomic charges are a useful and intuitive concept for understanding molecular properties and chemical reaction mechanisms, showing how changes in molecular geometry can affect the flow of electronic charge within a molecule. Both basis sets and exchange-correlation functional are important in atomic charge population analysis.[3] The use of partial atomic charge helps in the characterization of ground and excited-state electronic structure.[4] Here, we used Lowdin population analysis to study the ground state of the system in the absence of constraints. The results are tabulated for two different model systems: we analyze the Lowdin charges for model (I), graphene & adsorbed molecule, and model (II) which consists of graphene & water & adsorbed molecule. In model (II) the remainder of the partial charge exists on the water molecules. Normal DFT calculations show that the ground states for DMDQ, OH-Vi, and Me-Vi cases are a +1 charge on molecules and a -1 charge on graphene. While for dBR5 and 2HNQ, graphene retains -2 electrons.

Table S1: Lowdin population charges computed for model (I), graphene & adsorbed molecule, model (II) that consists of graphene & water & adsorbed molecule. $q_X(e)$, $q_G(e)$, $q_{total}(e)$ denote total charges on the molecules, graphene, and whole system, respectively. In model (II), the missing charge is located on water molecules.

	model (I)		model (II)		
Molecule	$q_X(e)$	$q_G(e)$	$q_X(e)$	$q_G(e)$	$q_{total}(e)$
DMDQ	0.83	-0.83	1.04	-0.51	0
OH-Vi	0.74	-0.74	1.35	-0.36	0
Me-Vi	0.90	-0.90	0.99	-0.63	0
dBR5	-0.04	-1.96	0.28	-1.12	-2
2HNQ	-0.63	-1.37	-0.56	-0.33	-2

3 Redox potential in solution (homogeneous) phase

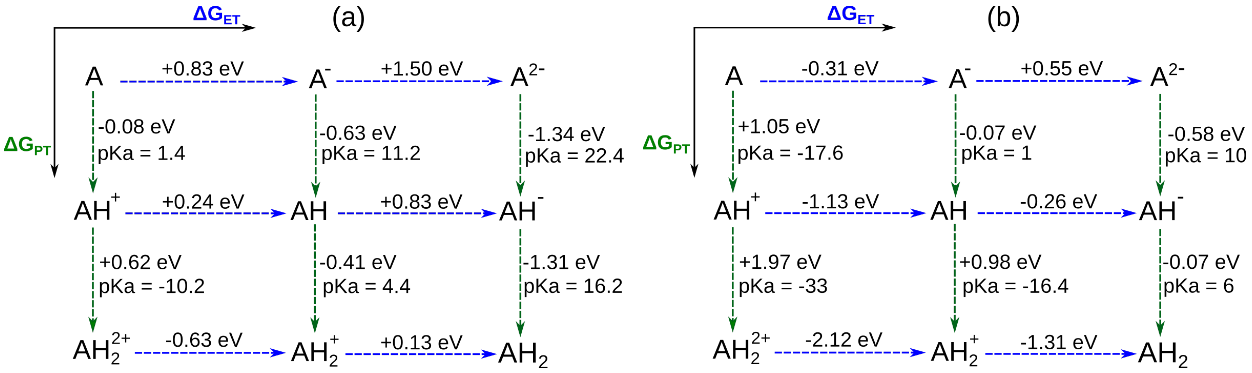

Figure S1: Square representation for a two-proton two-electron transfer in the reduction reaction of (a) dBR5 and (b) 2HNQ at pH = 0. A standard Hydrogen electrode (SHE) is considered as the reference point for the redox reactions.

The redox properties of the considered molecules can be described as a proton-coupled electron transfer (PCET) where the electron and proton transfer simultaneously or by a decoupled sequence of an electron transfer (ET; blue arrows in Figure S1) and a proton transfer (PT; green arrows in Figure S1) step. Readers interested in this topic are referred to the literature.[5,6] Combining these possible reaction paths results in a "scheme of squares" which is commonly used to evaluate and represent their redox properties (Figure S1).

First, we aim to show that the protonation steps are unavoidable at a range of pH. Therefore, we focus on the energetics of the decoupled electron and proton transfer reactions. Both dBR5 and 2HNQ molecules are of the A type and thus, be fully reduced by taking two electrons and protons in the process:

$$\mathrm{A} + 2\mathrm{H}^+ + 2\mathrm{e}^- \rightleftharpoons \mathrm{AH}_2. \tag{S14}$$

The energetics of the ET step, e.g. for $\mathrm{A} + \mathrm{e}^- \rightleftharpoons \mathrm{A}^-.$, can be straightforwardly computed as:

$$\Delta G_{\mathrm{ET}} = G(\mathrm{A}^-) - G(\mathrm{A}) - G_{e^-}, \tag{S15}$$

Where, $G(\mathrm{A}^-)$ and $G(\mathrm{A})$ correspond to the Gibbs free energies of the reduced species and the oxidized states, respectively. G_{e^-} indicates the Gibbs free energy of an electron.

Similarly, the energetics of PT steps, e.g. for $\mathrm{A} + \mathrm{H}^+ \rightleftharpoons \mathrm{AH}^+$., can be defined as

$$\Delta G_{\mathrm{PT}} = G(\mathrm{AH}^+) - G(\mathrm{A}) - G_{\mathrm{H}^+}. \tag{S16}$$

Here, $G(\mathrm{AH}^+)$ corresponds to the Gibbs free energies of the protonated species, and G_{H^+} represents a proton's Gibbs free energy.

In order to compute the reaction of the PT and ET, we need to know the Gibbs free energy of H^+ and e^-, respectively. The energetics of the PT are computed using the isodesmic method which employs the experimental pK_a of a reference compound (p$K_a^{ref.}$).[7,8] Herein, formic acid (HCOOH) dissociation reaction with p$K_a^{ref.} = 3.77$ is employed as a reference:[9]

$$\mathrm{HCOOH} \Longrightarrow \mathrm{HCOO}^- + \mathrm{H}^+. \tag{S17}$$

The pK_a value is defined as:

$$\mathrm{p}K_a = -\frac{\Delta G_{\mathrm{reaction}}}{RTln(10)}, \tag{S18}$$

where R and T are the general gas constant and temperature, respectively. In addition, $\Delta G_{\mathrm{reaction}}$ indicates the difference between reactant and product Gibbs free energy. Using Eq. S18, the value of $\Delta G_{\mathrm{reaction}}$ and, consequently, G_{H^+} are determined ($G_{\mathrm{H}^+} = G_{\mathrm{reaction}} + G_{\mathrm{HCOOH}} - G_{\mathrm{HCOO}^-}$). Therefore, the p$K_a$ value can be calculated for all the considered reactions.

We already know that cost of a proton used for redox reaction occurred in an implicit solvation model. Considering standard hydrogen electrode (SHE) as a reference electrode which is based on:

$$2\mathrm{H}^+(aq.) + 2e^- \Longrightarrow \mathrm{H}_2(g), \tag{S19}$$

the electron's Gibbs free energy is calculated as:

$$G_{e^-} = \frac{1}{2} G_{H_2(g)} - G_{H^+}. \tag{S20}$$

Here, $G_{H_2(g)}$ denotes the Gibbs free energy of H_2 in the gas phase.

Generally, researchers use a value of −4.44 eV as the Gibbs free energy of an electron. It is the International Union of Pure and Applied Chemistry[10] (IUPAC) recommendation for the potential of an electron in a vacuum compared to SHE, i.e. +4.44 V. We know that this quantity depends on the xc-functional, basis sets, and implicit solvent models employed.[11] In our calculation setup, the required Gibbs free energies were obtained by converging all the structures using PBE[12] exchange-correlation functional in combination with the PCM implicit solvation model[13,14] and a double zeta basis set of Def2-TZVP,[15] as implemented in Gaussian 16 revision C.01.[16] software.

We calculate the Gibbs free energies of HCOOH and $HCOO^-$ compounds in the implicit water and H_2 in the gas phase. In order to determine G_{H^+} and G_{e^-} values, we use equations (S17), (S18), and (S19). Our calculation setup results in a value of −12.26 and −3.62 eV for G_{H^+} and G_{e^-}, respectively. To compute onset potential and pK_a values, we first calculate the Gibbs free energies of reactant and product agents in the implicit water. Then, we compute reaction-free energy for ET and PT using equations (S15) and (S16), respectively. Finally, we define onset potential $E^0_{sol.}$, which corresponds to the required potential to involve only an electron in reaction, as:

$$E^0_{sol.} = -\frac{\Delta G_{reaction}}{e}. \tag{S21}$$

and pK_a as Eq. S18. Additionally, the most stable tautomers were identified by computing the energies of the different structures where needed.

4 Marcus theory regions and adiabatic free energy surfaces

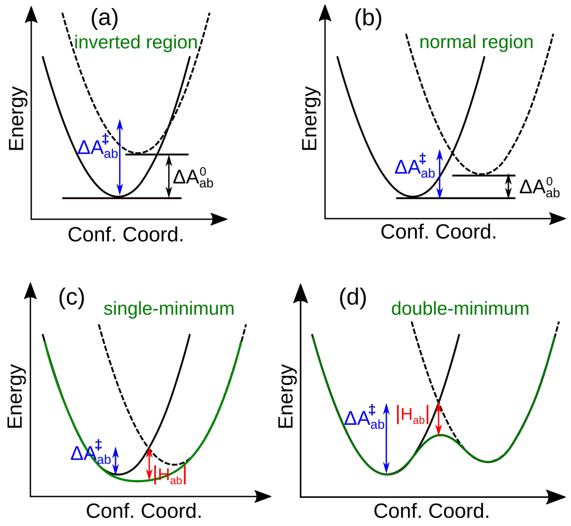

Figure S2: Profile of free energy surface of redox reactants (solid line) and that of products (dashes line) schematized versus configurational coordinate of all atoms in the system. The Electrochemical redox reaction occurs at (a) Marcus normal region and (b) Marcus inverted region. The adiabatic surfaces are indicated by green lines for the case of (c) a single-minimum energy surface and (d) a double-minimum energy surface. $\Delta A^{\ddagger}_{ab}$, $|H_{ab}|$, and ΔA^{0}_{ab} indicate the reduction reaction activation barrier free energy, diabatic electronic coupling, and reduction reaction free energy, respectively.

Since the nuclei do not have time to move during the quick electron jump, the two new species created when an electron jumps from one reaction species to another are in the incorrect environment of the solvent molecules and chemical structure of the reactants. The system reaches the ground state of the product after fluctuations (nuclei relaxation) take place. In Figure S2, a profile of the free energy surface is provided for several instances. The abscissa is a multidimensional configuration coordinate. Two parabolas represent the energy profile of the reactant (solid line) and products (dashed line). Overall, the electron transfer takes place in an inverted Marcus region if the crossing point of two parabolas happens outside of the two minima range (see Figure S2 (a)). Contrarily, it occurs in the Normal region (see Figure S2 (b)). Single-minimum energy surface appears when the electronic

interaction is strong enough to be greater than the activation barrier-free energy (see Figure S2 (c)). If not, a double-minimum energy profile is anticipated (see Figure S2 (d)).

5 Snapshot taken along the CDFT-AIMD

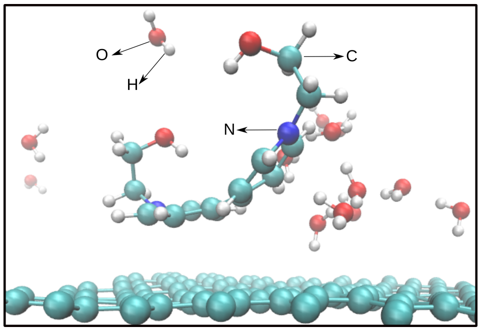

Figure S3: Snapshot from CDFT-AIMD simulations when neutral OH-Vi molecule stands on top of neutral graphene.

It appears that water molecules are attempting to peal the OH-Vi out of the graphene after around 1.5×10^3 steps of CDFT-AIMD simulations for neutral graphene and the OH-Vi molecule. One snapshot is represented in Figure S3.

6 Finite-size effects and scheme of supercell extension

A significant change in the Fermi-level energy in smaller supercells with respect to their charge state is shown in Figure S4 (a). As N, the number of carbon atoms in the supercell, increases, we observe that the variation decreases. Therefore, for the electrode to maintain a constant potential, the size must be infinite. However, we utilize a supercell of 15×9 unit cell containing 540 carbon atoms whose Fermi-level energy varies less than 130 meV between two successive charge states. In Figure S4 (b) and (c), the ΔA^0_{IJ} and λ values show systematic increases and decreases compared to the 7×4 graphene supercell, respectively. Those 15×9 graphene supercells are generated by embedding carbon atoms into the graphene from the 7×4 resulting CDFT-AIMD trajectories, as shown in Figure S5. While the atoms from step

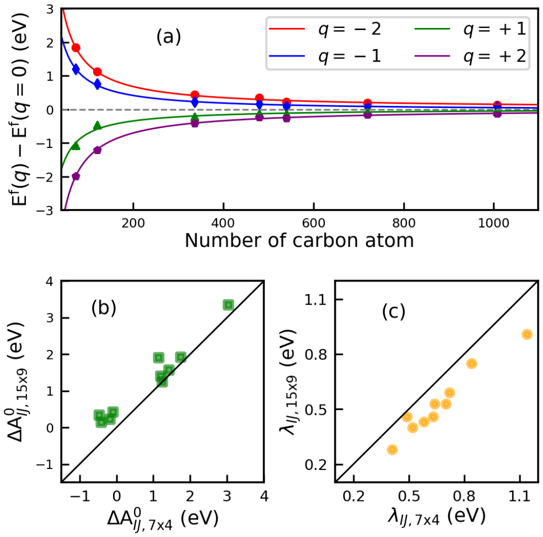

Figure S4: (a) Fermi-level energy of graphene at different charges (q) versus the number of carbon atoms. Reference is made to neutral graphene. Parity plots of (b) reaction free energy (ΔA^0_{IJ}) and (c) reorganization free energy (λ) evaluated by 15x9 graphene (540 C atoms) and 7×4 graphene (112 C atoms).

the latter calculations are frozen, the geometry of the added carbon atoms is optimized using DFT.

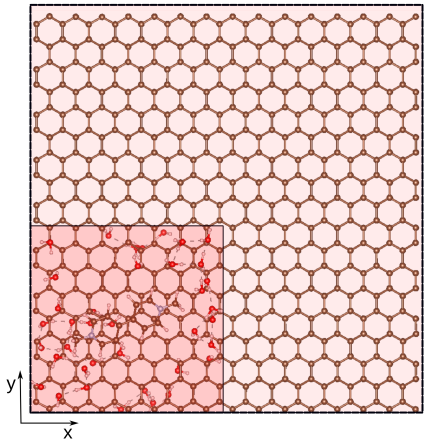

Figure S5: Geometry of a 7×4 graphene (inner supercell) containing explicit water is increased to 15x9 by patching graphene unit cell.

References

(1) Zener, C.; Fowler, R. H. Non-adiabatic crossing of energy levels. *Proceedings of the Royal Society of London. Series A, Containing Papers of a Mathematical and Physical Character* **1932**, *137*, 696–702.

(2) Landau, L. D.; Lifshitz, E. M. *Quantum Mechanics: Non-relativistic Theory. V. 3 of Course of Theoretical Physics*; Pergamon Press, 1958.

(3) Brehm, M.; Thomas, M. Optimized Atomic Partial Charges and Radii Defined by Radical Voronoi Tessellation of Bulk Phase Simulations. *Molecules* **2021**, *26*.

(4) MacDonell, R. J.; Patchkovskii, S.; Schuurman, M. S. A Comparison of Partial Atomic Charges for Electronically Excited States. *Journal of Chemical Theory and Computation* **2022**, *18*, 1061–1071, PMID: 35015528.

(5) Tyburski, R.; Liu, T.; Glover, S. D.; Hammarström, L. Proton-Coupled Electron Transfer Guidelines, Fair and Square. *Journal of the American Chemical Society* **2021**, *143*, 560–576, PMID: 33405896.

(6) Koper, M. T. M. Theory of multiple proton–electron transfer reactions and its implications for electrocatalysis. *Chem. Sci.* **2013**, *4*, 2710–2723.

(7) Ho, J.; Coote, M. L. A universal approach for continuum solvent pKa calculations: are we there yet? *Theoretical Chemistry Accounts* **2009**, *125*, 3.

(8) Ho, J. Predicting pKa in Implicit Solvents: Current Status and Future Directions. *Aust. J. Chem.* **2014**, *67*, 1441–1460.

(9) Ho, J.; Coote, M. L. A universal approach for continuum solvent pKa calculations: are we there yet? *Theoretical Chemistry Accounts* **2009**, *125*, 3.

(10) Currie, L. A. Nomenclature in evaluation of analytical methods including detection and quantification capabilities (IUPAC Recommendations 1995). *Pure and Applied Chemistry* **1995**, *67*, 1699–1723.

(11) Busch, M.; Ahlberg, E.; Ahlberg, E.; Laasonen, K. How to Predict the pKa of Any Compound in Any Solvent. *ACS Omega* **2022**, *7*, 17369–17383.

(12) Perdew, J. P.; Burke, K.; Ernzerhof, M. Generalized Gradient Approximation Made Simple. *Phys. Rev. Lett.* **1996**, *77*, 3865–3868.

(13) Scalmani, G.; Frisch, M. J. Continuous surface charge polarizable continuum models of solvation. I. General formalism. *The Journal of Chemical Physics* **2010**, *132*, 114110.

(14) Mennucci, B.; Cammi, R.; Tomasi, J. Excited states and solvatochromic shifts within a nonequilibrium solvation approach: A new formulation of the integral equation formalism method at the self-consistent field, configuration interaction, and multiconfiguration self-consistent field level. *The Journal of Chemical Physics* **1998**, *109*, 2798–2807.

(15) Weigend, F.; Ahlrichs, R. Balanced basis sets of split valence, triple zeta valence and quadruple zeta valence quality for H to Rn: Design and assessment of accuracy. *Phys. Chem. Chem. Phys.* **2005**, *7*, 3297–3305.

(16) Frisch, M. J.; Trucks, G. W.; Schlegel, H. B.; Scuseria, G. E.; Robb, M. A.; Cheeseman, J. R.; Scalmani, G.; Barone, V.; Petersson, G. A.; et al., H. N. Gaussian 16 Revision C.01. 2016.
